# Differences in standing behavior between Jersey and Holstein dairy cows during the periparturient period

**DOI:** 10.3168/jdsc.2023-0502

**Published:** 2024-03-02

**Authors:** Lauren V. Hare, Marie-Claire Lamoureux, Juliana M. Huzzey

**Affiliations:** Department of Animal Science, California Polytechnic State University, San Luis Obispo, CA 93407

## Abstract

Jerseys are the second most popular breed in the US dairy industry, yet there are few studies that directly compare their behavior with Holsteins. The objective of this observational study was to describe differences in the standing behavior of healthy Jersey and Holstein dairy cows during the periparturient period. A total of 51 Jerseys (11 primiparous [PP] and 40 multiparous [MP]) and 36 Holsteins (8 PP, 28 MP) were used for this analysis. Data loggers that measured leg orientation were used to determine daily standing time (min/d), number of standing bouts (no./d), and standing bout duration for each cow from approximately 3 wk before until 4 wk after calving. Holstein and Jersey cows were commingled throughout the periparturient period and only healthy cows were included in the analysis. Overall, Jerseys had longer standing times and longer standing bout durations compared with Holsteins during the period before calving. On the day before calving, PP cows had longer standing times than MP cows, but there was no effect of breed on standing behavior during the calving period (d −1, 0, and +1 relative to calving). The number of standing bouts for all cows spiked on the day of calving, but this increase was greatest for the MP cows, regardless of breed. Primiparous cows had longer postpartum standing times compared with MP cows; however, PP Jerseys tended to have the longest daily standing times during the first 10 DIM. After calving, there were no breed differences in the number of standing bouts per day among MP cows; however, PP Jerseys had more standing bouts than PP Holsteins during this time. Understanding how Jersey and Holstein behaviors differ may offer insights into better management of Jerseys during the period around calving.

## Highlights

• Jerseys had longer prepartum standing times and standing bout durations.
• Breed had no effects on standing behavior during the calving period.
• After calving, multiparous Jersey and Holstein cows had similar standing behavior.
• Primiparous Jerseys had more postpartum standing bouts than primiparous Holsteins.
• Regardless of breed, primiparous cows had longer peripartal standing times.

## Graphical Abstract

**Figure:**
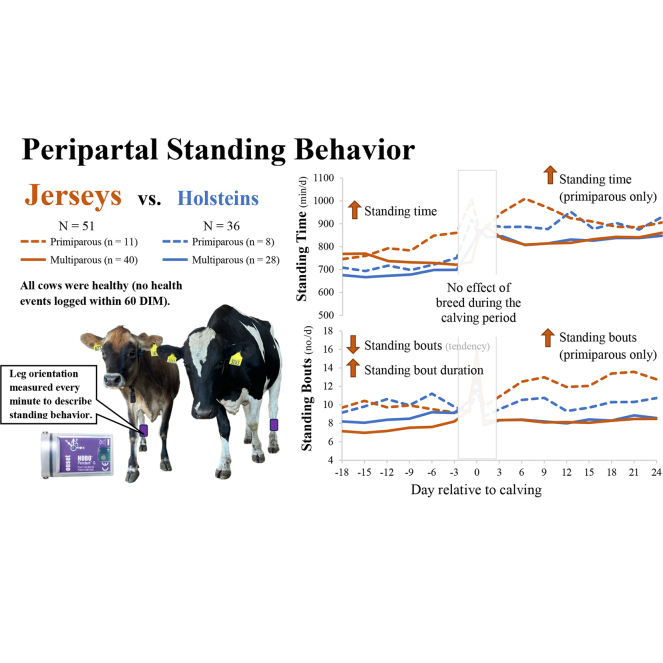
**Summary** Few studies have directly compared the behavior of Jerseys with Holsteins. Our objective was to compare the standing behavior of healthy Jersey and Holstein cows around calving. Fifty-one Jerseys (11 primiparous [PP], 40 multiparous [MP]) and 36 Holsteins (8 PP, 28 MP) were studied. Data loggers monitored standing behavior from 3 weeks before until 4 weeks after calving. Before calving, Jerseys spent more time standing each day and had longer standing bout durations than Holsteins. After calving, regardless of breed, PP cows had longer standing times and more standing bouts each day than MP cows. Postpartum standing behavior of MP Jersey and Holstein cows was similar; however, PP Jerseys had more standing bouts than PP Holsteins during this time. Understanding how Jersey and Holstein behaviors differ may offer insights into better management of Jersey cows during the period around calving.

In the US dairy industry, Holsteins represent 86% of the population, with the next most common breed being Jerseys, which constitute 8% of the industry (USDA, 2016). The Jersey is a popular breed for various reasons, one of which being their higher milk fat and protein percentage relative to Holsteins (CDBC, 2021). Jerseys have also been reported to have better production efficiency (defined as milk solids per DMI) compared with Holsteins, which is attributed in part to their larger gastrointestinal tract and different gut microbial population, which improves feed digestibility (Beecher et al., 2014). Age at first calving is lower in Jerseys, as is their calving interval compared with other breeds (Hare et al., 2006). Jerseys are also less likely to experience dystocia during calving (Dhakal et al., 2013).

Although Jerseys represent a significant portion of the dairy industry, there has been a notable lack of research comparing their behavior with that of Holsteins. Moreover, existing publications on this subject have yielded inconsistent findings. For instance, Prendiville et al. (2010) found no differences in grazing time, the number of grazing bouts, grazing bout duration, or rumination time between lactating Jersey and Holstein cows managed on pasture. Similarly, Stone et al. (2017) reported no effect of breed on the rumination time of lactating cows housed indoors. In contrast, Munksgaard et al. (2020), who studied both breeds in an indoor loose-housing system with electronic feeders (Insentec B.V., Marknesse, the Netherlands) and an automated milking system, found that Jerseys spent less time eating compared with Holsteins throughout their lactation. Munksgaard et al. (2020) also reported that Jerseys spent less time lying and took more steps per day compared with Holsteins; however, Stone et al. (2017) did not identify differences in lying time between these breeds.

An improved understanding of normal behavior in Jerseys may provide insights into better on-farm management for this breed. An example of an application is in the use of behavior to identify or predict ill health during the transition period, work that has focused on Holsteins (Weary et al., 2009). Differences in the behavior of healthy Jersey and Holstein cows may mean that the relationships between behavior and disease between breeds are also different. To our knowledge, no research has directly compared differences in behavior between breeds throughout the periparturient period.

The objective of this study was to compare the standing behavior of healthy Jersey and Holstein dairy cows from approximately 3 wk before until 4 wk after calving. We hypothesized that Jerseys may have longer standing times, more standing bouts, and longer standing bout durations than Holsteins because (1) standing is more comfortable for a less heavy animal (reduced pressure on feet and less energy needed to change body position), or (2) because they are more socially subordinate to the larger Holsteins, and thus have difficulty maintaining access to resources such as the feed bunk. Alternatively, Jerseys may not need to spend as much time at the feed bunk due to their lower DMI and milk yield, which would support the hypothesis that their standing times after calving may be shorter than Holsteins.

This observational study was designed as a cohort study. Holstein and Jersey cows were monitored through the periparturient period, and their standing behavior was compared retrospectively after determining their health status. Data were collected at the California Polytechnic State University (San Luis Obispo, CA) Dairy Unit between June 2016 to June 2017 and June 2022 to July 2023, and animal use was reviewed and approved by the Institutional Animal Care and Use Committee (protocol ID #1517 and #2205).

Management of cows during the periparturient period remained consistent during both observation periods. At the time of enrollment, cows were housed in a far-off dry cow pen that consisted of a freestall barn with access to an outdoor dry lot with groomed composted manure. Approximately 3 wk before the cows expected calving date, they were moved to a close-up pen, which consisted of an indoor and outdoor bedded-pack area with composted manure. While in the far-off and close-up pens, heifers and cows were housed in separate groups, but both breeds were housed together. Cows calved in the close-up pen and then were moved within 12 h of calving to a neighboring transition pen. The layout of the transition pen was the same as the close-up pen; however, the group composition consisted of both primiparous and multiparous cows within 1 to 3 DIM and both breeds. Cows that were healthy at 3 DIM were moved to a single lactation pen that housed both primiparous and multiparous cows and both breeds. The lactation pen consisted of a freestall barn with access to an outdoor dry lot with groomed composted manure. Shades were not provided in the outdoor dry lots, but cows could move freely between the covered barn and outdoor area. All pens used a headlock feed barrier. Stocking density was variable throughout the data collection period and data on stocking rates was not maintained; however, farm management protocols aimed to not exceed an 80% stocking rate in the close-up, transition, and lactation pens. Cows were fed a TMR twice daily at approximately 0500 and 1600 h. The prepartum and postpartum TMR consisted of a combination of forage (alfalfa hay, grass hay, grass silage, and corn silage), wet brewers grain, concentrate grain (corn grain, soybean meal, canola meal, whole cottonseed, and wheat middlings), and mineral mix, formulated in proportions appropriate for dry and lactating cows. Lactating cows were milked twice daily in a parallel milk parlor at approximately 0600 and 1700 h.

Cows were eligible for enrollment 3 wk before their expected calving date if they had no clinical signs of illness. At enrollment, a data logger (HOBO Pendant G Acceleration Data Logger, Onset Computer Corporation, Pocasset, MA) was attached to the cow's hind leg to monitor standing activity. The logger was wrapped to the leg using elastic bandages and changed weekly to download the data and switch the logger position to the alternate leg. The logger recorded leg orientation at 1-min intervals and this information was analyzed by a SAS algorithm developed by the UBC Animal Welfare Program (2013) to provide data on daily standing time (min), daily number of standing bouts, and daily standing bout duration (min). Standing behavior was monitored continuously throughout the periparturient period until 28 d postpartum. Health status of all cows was monitored by the farm staff, and any occurrence of mastitis, metritis, ketosis, lameness, milk fever, displaced abomasum, retained placenta, or other miscellaneous health events (e.g., injury, died, dystocia) were noted in the herd management system. Health records were evaluated to select a final population of healthy animals to include in the analysis. A cow was considered healthy if it had no health event logged within the 60-d period following calving, with one exception: if a cow had an incident of retained placenta but no further health event logged (n = 4), the cow was included in the analysis. Monthly average temperature and humidity data were obtained from local weather station records (NOAA, 2023), and temperature-humidity index (**THI**) was calculated following the methods of Stone et al. (2017). This information was used to determine whether cows calved in the cool season (THI <68) or warm season (THI ≥68). The maximum temperature, minimum temperature, and percent humidity (mean ± SD) during the warm months were 25 ± 2°C, 11 ± 2°C, and 61 ± 4%, respectively, and during the cool months were 18 ± 2°C, 7 ± 2°C, and 64 ± 3%, respectively.

From 2016 to 2017, a total of 115 cows were initially enrolled into the study and 51 healthy cows were used for subsequent data analyses. Of the 64 cows excluded, 30 were due to health reasons (mastitis [7], metritis [2], ketosis [2], milk fever [3], displaced abomasum [2], lame [10], and other [4]), 8 were due to missing or incomplete health data, and 26 were due to missing data logger data (calved early [<14 d of prepartum data] or logger stopped working for >7 consecutive days). From 2022 to 2023, a total of 83 cows were enrolled into the study and 36 healthy cows were used for subsequent data analyses. Of the 47 cows excluded, 45 were due to health reasons (mastitis [6], metritis [11], ketosis [17], milk fever [2], displaced abomasum [2], and other [7]), and 2 were excluded due to missing data logger data.

Overall, a total of 51 healthy Jersey cows (11 primiparous [**PP**] and 40 multiparous [**MP**]) and 36 healthy Holstein cows (8 PP and 28 MP) were included in the analyses. All analyses were performed using SAS (version 9.4, SAS Institute Inc.), and cow (n = 87) was considered the experimental unit. For each cow, daily estimates of standing time, standing bouts, and standing bout duration were pooled into 6 periods before calving and 8 periods after calving. Each period was based on an average of 3 d, with the mid-point (day relative to calving) of the 3-d average used to describe the period (e.g., period −3 = d −4, −3, and −2 relative to calving). Prepartum (periods: d −18, −15, −12, −9, −6, and −3 relative to calving) and postpartum (periods: d 3, 6, 9, 12, 15, 18, 21, and 24 relative to calving) data were analyzed in independent models. Differences in pre- and postpartum standing time, standing bouts, and standing bout duration between breeds were analyzed using PROC MIXED; the models included the fixed effects of calving season (warm vs. cool), parity (PP vs. MP), breed, period (d −18 to −3 and d 3 to 27, analyzed in separate models), and the interactions: parity × breed, season × breed, parity × period, breed × period, and parity × breed × period. To analyze how standing time, standing bouts, and standing bout duration differed between breeds during the calving period, we used a mixed model that included the fixed effects of calving season, parity, breed, day (−1, 0, and +1 relative to calving) and the interactions: parity × breed, season × breed, parity × day, breed × day, and parity × breed × day. Period and day were considered repeated measures, and an autoregressive covariance structure was used based on best fit using the Bayesian information criterion. Cow was included in the models as a random effect. Standing bout duration was log-transformed for analysis; the LSM and CI were back-transformed to report the geometric mean and 95% CI on the original scale.

Before calving, Jerseys spent more time standing than Holsteins (774 ± 13 vs. 699 ± 16 min/d, *P* < 0.001; Figure 1A) and PP cows spent more time standing than MP cows (759 ± 17 vs. 714 ± 9 min/d, *P* = 0.02). The daily standing time of PP cows increased as they neared parturition, whereas the standing time of MP cows remained relatively steady (parity × period interaction, *P* = 0.05). Cows that calved during the warm season spent less time standing during the prepartum period than cows that calved during the cool season (710 ± 11 vs. 763 ± 15 min/d, *P* = 0.002), and this effect was more pronounced in Holsteins compared with Jerseys (breed × season interaction, *P* = 0.07). Standing time during the calving period was not influenced by breed (*P* = 0.57); however, parity did have an influence (*P* = 0.01). Primiparous cows spent more time standing the day before calving, compared with d 0 and +1, whereas MP cows spent less time standing the day before calving, compared with d 0 and +1 (parity × period interaction, *P* < 0.001; Figure 1A). Cows that calved during the warm season spent less time standing during the calving period than cows that calved during the cool season (844 ± 16 vs. 901 ± 24 min/d, *P* = 0.03). During the postpartum period, PP cows had longer daily standing times than MP cows (918 ± 17 vs. 837 ± 9 min/d, *P* < 0.001; Figure 1A). A parity × period (*P* = 0.01) and breed × period (*P* = 0.01) interaction revealed that the standing time of PP cows and Jersey cows declined across the postpartum periods, respectively; this decline tended to be influenced by the PP Jerseys who had the highest standing times during the days following calving (parity × breed × period interaction, *P* = 0.09). Calving season did not influence postpartum standing time (*P* = 0.11).

**Figure 1:**
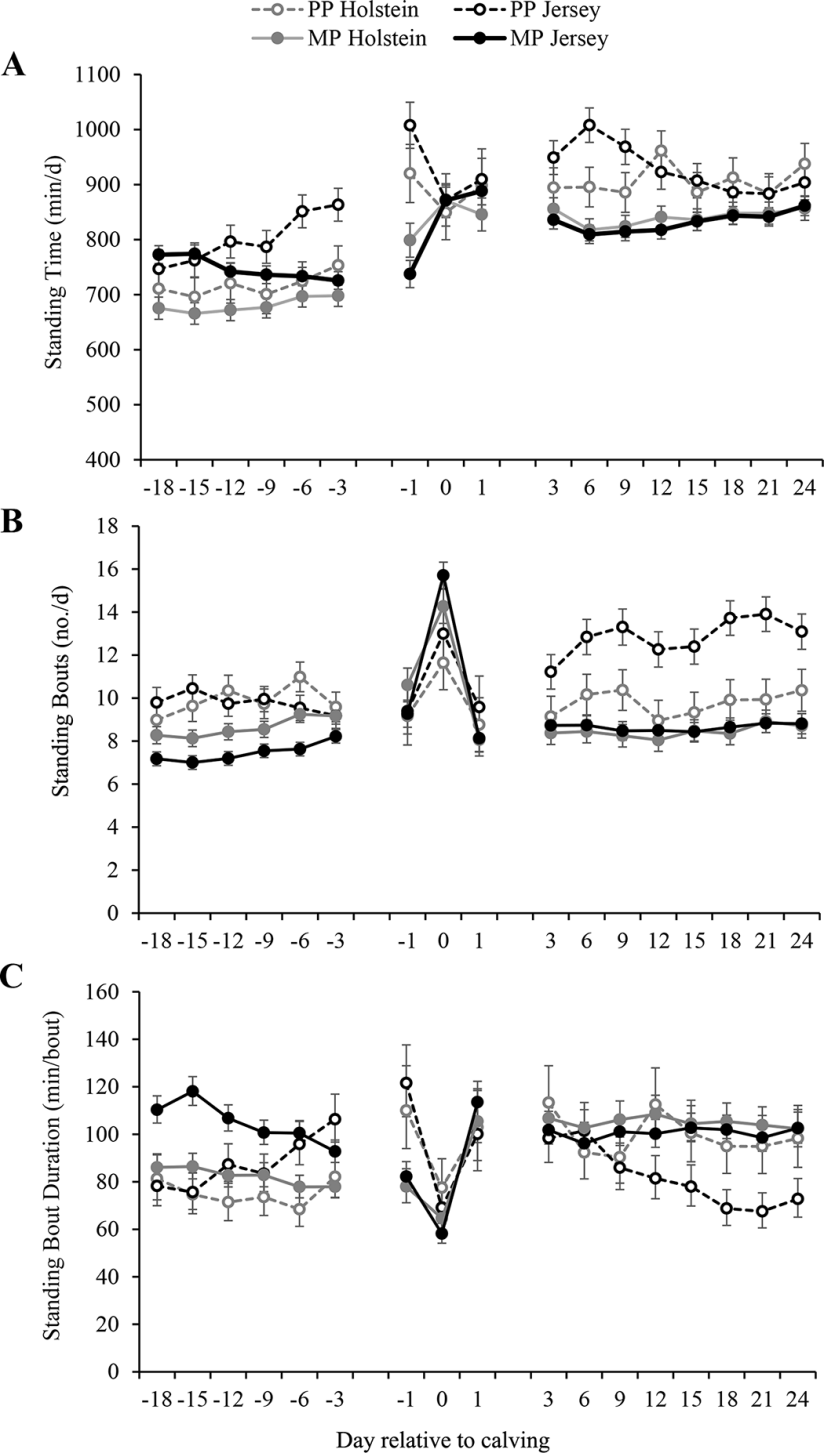
Least squares means (±SE) of standing time (A; min/d), LSM (±SE) of standing bouts (B; no./d), and back-transformed LSM (±95% CI) of standing bout duration (C; min/bout) for primiparous (PP) and multiparous (MP) Jersey and Holstein cows during the periparturient period. Data points during the prepartum (d −18 to −3) and postpartum (d 3 to 24) periods are based on an average of 3 d, with the mid-point (day relative to calving) of the 3-d average reported on the x-axis. Data within these 3 time periods were analyzed independently and are represented by the broken line.

During the prepartum period, PP cows had more standing bouts per day than MP cows (9.8 ± 0.3 vs. 8.0 ± 0.2 bouts/d, *P* < 0.001) and Holsteins tended to have more standing bouts per day than Jerseys (9.3 ± 0.3 vs. 8.6 ± 0.2 bouts/d, *P* = 0.09; Figure 1B). Among Holsteins, the number of standing bouts per day increased as parturition approached, but this increase was not observed in Jerseys (breed × period interaction, *P* = 0.05). Multiparous cows also tended to increase their number of daily standing bouts as parturition approached compared with PP cows (breed × parity interaction, *P* = 0.07). Cows that calved during the warm months tended to have more standing bouts during the prepartum period than cows that calved during cool months (9.2 ± 0.2 vs. 8.7 ± 0.3 bouts/d, *P* = 0.08). The number of standing bouts peaked for all cows on the day of calving (*P* < 0.001), and this change was greatest for the MP cows (parity × period interaction, *P* = 0.03; Figure 1B). There were no effects of calving season (*P* = 0.24) or breed (*P* = 0.53) on the number of standing bouts during the calving period. After calving, PP cows had more standing bouts per day than MP cows (*P* < 0.001). A breed × parity interaction (*P* = 0.003) showed that differences in the number of standing bouts between breeds were restricted to the PP cows (Figure 1B). After calving, PP Jerseys had more standing bouts than PP Holsteins (12.5 ± 0.6 vs. 10.1 ± 0.6 bouts/d); however, the number of daily standing bouts did not differ between MP Holstein and MP Jersey cows (8.4 ± 0.4 vs. 8.6 ± 0.3 bouts/d, respectively). The number of daily standing bouts increased over time during the postpartum period (*P* = 0.02) and this increase tended to be more pronounced in the PP cows compared with the MP cows (parity × period interaction, *P* = 0.07). During the postpartum period, differences in the number of standing bouts between Holsteins and Jerseys that calved during the cool season (8.7 ± 0.6 vs. 11.7 ± 0.4 bouts/d, respectively) were greater than differences observed between Holsteins and Jersey that calved during the warm season (9.4 ± 0.4 vs. 9.8 ± 0.3 bouts/day, respectively; calving season × breed interaction, *P* = 0.003).

During the prepartum period, Jerseys had longer standing bout durations than Holsteins (95 min/bout [95% CI: 91–99] vs. 79 min/bout [95% CI: 75–82]; *P* = 0.002, Figure 1C) and PP cows had shorter standing bout durations than MP cows (81 min/bout [95% CI: 77–85] vs. 93 min/bout [95% CI: 90–95]; *P* = 0.01). A parity × period interaction (*P* = 0.01) revealed that the average standing bout duration of PP cows increased as parturition approached but decreased among MP cows. Cows that calved during the warm season had shorter standing bout durations during the prepartum period than cows that calved during the cool season (*P* = 0.003). There was no effect of breed on average standing bout duration during the calving period (*P* = 0.97). Primiparous cows had longer standing bout durations than MP cows on the day before calving; however, by d 0 and +1, standing bout durations were similar between these groups (parity × period interaction, *P* = 0.03). During the calving period, average standing bout duration tended to be shorter for cows that calved during the warm season compared with those that calved during the cool season (*P* = 0.07). After calving, PP cows had shorter standing bout durations than MP cows (90 min/bout [95% CI: 84–95] vs. 103 min/bout [95% CI: 99–106]; *P* = 0.05) and Jerseys tended to have shorter standing bout durations than Holsteins (90 min/bout [95% CI: 86–95] vs. 102 min/bout [95% CI: 97–108]; *P* = 0.09). Differences in postpartum standing bout duration between Holsteins and Jerseys that calved in the cool season (110 min/bout [95% CI: 101–119] vs. 83 min/bout [95% CI: 77–89], respectively) were greater than differences observed between Holsteins and Jersey that calved in the warm season (95 min/bout [95% CI: 90–101] vs. 99 min/bout [95% CI: 93–104], respectively; calving season × breed interaction, *P* = 0.01).

This study found that Jerseys had longer standing times, fewer standing bouts, and longer standing bout durations compared with Holsteins during the period before calving; this may be attributed to body size of cow and calf. Holsteins produce larger calves than Jerseys (Dhakal et al., 2013), which could make lying more uncomfortable and cause Holsteins to change their body position more often. Although there is a lack of direct research describing the relationship between standing behavior and BW (Tucker et al., 2021), it seems reasonable to predict that long standing times are uncomfortable for heavier cows due to added pressure placed on their hooves and joints. It is possible that the influence of body size on standing behavior is better noticed during the prepartum period because the behavior is less confounded by management (e.g., time milk parlor) and physiology (e.g., longer feeding times to support the energy demands of lactation).

Differences in postpartum standing behavior between Jerseys and Holsteins, reported in the current study, differ from the results reported by Munksgaard et al. (2020); this may be due to differences in the way cows were managed after calving. Munksgaard et al. (2020) grouped Holsteins and Jerseys in separate pens, used an automated milking system, and housed cows in a freestall barn. In contrast, cows in the present study were managed as one group after calving, milked in a parlor twice daily, and provided free access to a dry lot in addition to the freestall barn. Munksgaard et al. (2020) reported that regardless of parity, Jerseys stood longer than Holsteins after calving; however, in the current study, the postpartum standing behavior of MP Jersey and Holstein cows was very similar. Jersey and Holstein cows were moved as one group to the milk parlor, and so regardless of their level of milk production, they would have spent the same amount of time away from the pen, standing to be milked. Cows managed on dry lots and deep-bedded packs do not need to compete for lying locations, and research shows they stand for longer periods of time compared with cows housed indoors (Tucker et al., 2021), which may be attributed to the softer surfaces that likely make standing more comfortable. In the present study, the 2 breeds may have also more closely synchronized their behavior because they were housed together. These factors may explain why we observed more similar standing behavior between older Jersey and Holstein cows compared with Munksgaard et al. (2020).

Our findings are consistent with several other studies that have found that PP cows have longer standing times (Barraclough et al., 2020; Munksgaard et al., 2020) and more lying bouts (Neave et al., 2017) during the periparturient period compared with MP cows. Differences in standing and lying behavior between PP and MP cows around calving may be a consequence of PP cows being lower in the social hierarchy relative to MP cows and thus less successful at competing for access to important resources such as the feed bunk or resting area. Primiparous cows may also be more disturbed by novel experiences (e.g., regrouping, commingling with older cows) associated with the periparturient period (Proudfoot and Huzzey, 2022). For example, Mazer et al. (2020) reported that when PP cows were regrouped alone into the lactation pen after calving, fecal cortisol metabolite concentrations (a physiological indicator of stress) were higher during the first 4 d after regrouping compared with regrouped MP cows. In the current study, it was the PP Jerseys that had the highest daily standing times, highest number of standing bouts, and shortest standing bout durations after calving, indicating that they may have had the greatest difficulty transitioning to a lactation pen. Primiparous Jerseys are physically the smallest animals in the pen and thus may have the most difficult time competing for, or maintaining access to, resources. However, body size alone cannot explain all differences in standing behavior after calving because standing behavior among the MP Holsteins and Jerseys was similar in the present study. Previous experience likely plays an important role in supporting a Jersey's ability to compete with larger Holsteins in a lactation pen; however, more research is needed to directly compare the links between standing, feeding, and social behaviors of Jerseys and Holsteins in commingled environments.

Cows that calved during the warm season had shorter standing times during the prepartum and calving periods than cows that calved during the cool season, and postpartum standing behavior was more variable between breeds during the cool months. These findings may have less to do with ambient temperatures, which even in the warm months remained relatively mild, and more to do with moisture, since the cool season was associated with increased rainfall. Studies have shown that cows will stand longer when it rains during winter months and will avoid being outside during rain (reviewed in Tucker et al., 2021). Although we did not keep track of where cows were lying in the current study, it is likely that cows spent more time lying in the freestalls during the cooler months due to rainy conditions, and this may have been less comfortable, explaining the longer standing times and more variable behavior.

In conclusion, the standing behavior of healthy Holstein and Jersey cows differs around calving. Future research should explore whether differences in standing behaviors between breeds are associated with differences in their ability to compete for access to resources. Our work provides some evidence that PP Jerseys have the most difficulty transitioning into a mixed parity and breed lactation pen, which raises the question of whether managing breed-specific groups would be beneficial during the periparturient period.
